# Electronic search strategies to identify reports of cluster randomized trials in MEDLINE: low precision will improve with adherence to reporting standards

**DOI:** 10.1186/1471-2288-10-15

**Published:** 2010-02-16

**Authors:** Monica Taljaard, Jessie McGowan, Jeremy M Grimshaw, Jamie C Brehaut, Andrew McRae, Martin P Eccles, Allan Donner

**Affiliations:** 1 Ottawa Hospital Research Institute, Clinical Epidemiology Program, Ottawa, Canada; 2 Department of Epidemiology and Community Medicine, University of Ottawa, Ottawa, Canada; 3 Faculty of Family Medicine, Faculty of Medicine, Ottawa, Canada; 4 Institute of Population Health, University of Ottawa, Ottawa, Canada; 5 Department of Information Studies, University of Aberystwyth, UK; 6 Department of Medicine, University of Ottawa, Ottawa, Canada; 7 Department of Epidemiology and Biostatistics, University of Western Ontario, London, Canada; 8 Institute of Health & Society, Newcastle University, Newcastle upon Tyne, UK; 9 Robarts Clinical Trials, London, Canada

## Abstract

**Background:**

Cluster randomized trials (CRTs) present unique methodological and ethical challenges. Researchers conducting systematic reviews of CRTs (e.g., addressing methodological or ethical issues) require efficient electronic search strategies (filters or hedges) to identify trials in electronic databases such as MEDLINE. According to the CONSORT statement extension to CRTs, the clustered design should be clearly identified in titles or abstracts; however, variability in terminology may make electronic identification challenging. Our objectives were to (a) evaluate sensitivity ("recall") and precision of a well-known electronic search strategy ("randomized controlled trial" as publication type) with respect to identifying CRTs, (b) evaluate the feasibility of new search strategies targeted specifically at CRTs, and (c) determine whether CRTs are appropriately identified in titles or abstracts of reports and whether there has been improvement over time.

**Methods:**

We manually examined a wide range of health journals to identify a gold standard set of CRTs. Search strategies were evaluated against the gold standard set, as well as an independent set of CRTs included in previous systematic reviews.

**Results:**

The existing strategy (randomized controlled trial.pt) is sensitive (93.8%) for identifying CRTs, but has relatively low precision (9%, number needed to read 11); the number needed to read can be halved to 5 (precision 18.4%) by combining with cluster design-related terms using the Boolean operator AND; combining with the Boolean operator OR maximizes sensitivity (99.4%) but would require 28.6 citations read to identify one CRT. Only about 50% of CRTs are clearly identified as cluster randomized in titles or abstracts; approximately 25% can be identified based on the reported units of randomization but are not amenable to electronic searching; the remaining 25% cannot be identified except through manual inspection of the full-text article. The proportion of trials clearly identified has increased from 28% between the years 2000-2003, to 60% between 2004-2007 (absolute increase 32%, 95% CI 17 to 47%).

**Conclusions:**

CRTs should include the phrase "cluster randomized trial" in titles or abstracts; this will facilitate more accurate indexing of the publication type by reviewers at the National Library of Medicine, and efficient textword retrieval of the subset employing cluster randomization.

## Background

The randomized controlled trial is widely accepted as the gold standard study design in health research [1]. In some situations, randomization of individuals is infeasible or undesirable, for example, because the intervention was designed to be administered at the cluster level (e.g., a mass media health promotion campaign) or because there is a risk of contamination when individuals in close proximity are allocated to competing interventions (e.g., a smoking cessation intervention in schools) [2]. In such cases, the presence of natural groups or social units nevertheless allows randomization to take place, albeit at the group level. Units of randomization in cluster randomized trials (CRTs) (also known as group randomized trials [3], community randomized trials, or place randomized trials [4]) are diverse and may include nursing homes, medical practices, hospital wards, schools, or villages.

In the present article, we focus on the problem of identifying CRT reports in the literature for the purpose of conducting systematic reviews. Although systematic reviews often focus on trials of a particular medical treatment or condition, several researchers have conducted systematic reviews of CRTs, to assess the methodological or reporting quality at various points in time [5-11]. Most reviewers have used hand-searching (or manual searching) of specific journals to identify CRT reports; for example, Donner et al. [5] used hand-searching of four medical and epidemiology journals to identify CRTs published between 1979 and 1989, after an electronic search failed to retrieve all relevant articles. Instead of hand-searching a selected set of journals, which may introduce bias into a systematic review, electronic searches may be implemented in online bibliographic databases such as MEDLINE, maintained by the US National Library of Medicine (NLM). MEDLINE, covering the fields of medicine, nursing, dentistry, veterinary medicine, the health care system, and the preclinical sciences, currently contains citations from approximately 5,200 international journals since 1949 to present [12]. Electronic search strategies ("filters" or "hedges") implemented in MEDLINE need to be acceptably sensitive (in that they retrieve a high proportion of articles relevant to the research) and precise (in that they do not retrieve a high proportion of articles irrelevant to the research). Simple electronic search strategies are available to easily identify reports of randomized controlled trials (for example, the Cochrane Collaboration's Highly Sensitive Search Strategy (2005 revision) [13]); however, the precision of such strategies for identifying CRTs is likely low as CRTs constitute a smaller subset of randomized controlled trials. The feasibility of implementing electronic searches especially targeted at CRTs depends on adequate reporting of the study design in the title or abstract; a description of the randomization units and procedures in the methods section of the article would not suffice as full-text searches of manuscripts are not currently possible in MEDLINE.

In recent years, authors and journal editors may have become more aware of the importance of adequately reporting the design of the study, especially after publication of the Consolidated Standards of Reporting Trials (CONSORT) statement [14], which includes a set of evidence-based guidelines and a checklist for reporting of trials. The CONSORT statement was adapted for CRTs in 2004 [15]; one of the recommendations was that authors clearly identify the trial as cluster randomized in the title or abstract. It is unknown to what extent authors and journal editors have adhered to these recommendations and whether there has been an improvement over time. Although there is currently no classification for "cluster randomized trial" in MEDLINE, adhering to the CONSORT recommendation should facilitate appropriate indexing of the publication type as "randomized controlled trial" and allow efficient text word retrieval of the subset of cluster randomized trials.

Our objectives in the present article are to (a) determine the sensitivity and precision of a simple, existing electronic search strategy for randomized controlled trials with respect to identification of CRTs, (b) determine the feasibility of alternative electronic search strategies incorporating cluster design-related terms, and (c) determine to what extent authors are appropriately identifying trials as cluster randomized in the titles or abstracts of reports and whether any improvement has occurred over time.

## Methods

### Identification of a gold standard set

We first identified a "gold standard" set of CRTs, by manually examining a total of 78 health journals indexed in MEDLINE between January 2000 and November 2007. All issues in a particular year, with the year assigned by computer-generated random numbers, were searched. Journals were purposely selected from a wide range of subject categories in the 2007 Sciences and Social Sciences editions of Journal Citation Reports (JCR), as well as based on our subjective knowledge of their likelihood to publish CRTs (see Additional file 1: "List of journals examined for the gold standard set"). Pilot studies, study protocols, methods papers, and studies using quasi-randomized designs were excluded, as well as articles reporting secondary results of a trial with main results published elsewhere. A subset of 10 major medical journals, including 7,584 articles all published during 2006, was initially examined independently by two reviewers to assess agreement in the identification of CRTs. Differences between reviewers were resolved by discussion. The kappa coefficient for initial agreement on inclusion of studies was 0.81 (95% confidence interval 0.71 to 0.90). These journals were then divided between the two reviewers to complete the manual searching. A total of 25,707 articles were examined, resulting in 162 reports of CRTs.

### Existing search strategy for randomized controlled trials

We evaluated a well-known existing search strategy for randomized controlled trials, involving a very brief search using the single publication type "randomized controlled trial". The *publication type *field is assigned by indexers at the NLM to classify the study type, for example, book reviews, abstracts, case reports, controlled clinical trials, and letters. This search strategy was originally labelled "simple strategy for the busy searcher" and was reported to be highly sensitive and precise in identifying randomized controlled trials in MEDLINE [13,16].

### Cluster design related search strategies

The full bibliographic details of all 162 CRTs in the gold standard set, including the title, abstract, Medical Subject Headings (MeSH), and publication type were exported to a statistical software package for analysis. From the title or abstract, we identified the specific text revealing the trial as cluster randomized (for example, "cluster randomized", "community randomized" or "group randomized") or possibly cluster randomized (for example, "hospitals were randomly assigned"). We conducted a frequency analysis of the exported text to identify candidate terms for building a search strategy.

### Evaluation of search strategies

Unique Identifier (UI) numbers, an 8-digit number assigned by the NLM to uniquely identify a particular record, were obtained for all 162 CRTs in the gold standard set and all 25,545 non-CRTs excluded from the gold standard set. Search strategies were implemented in the MEDLINE database (OVID interface) from 1996 to the third week of January 2009. We calculated the two performance indicators that are most relevant to systematic reviews [17], namely sensitivity, defined as the proportion of all the CRTs that are retrieved by a particular search; and precision, defined as the proportion of CRTs among the articles retrieved by a search strategy. Strategies with low precision place a greater burden on reviewers as more irrelevant articles have to be screened out; this is represented by 1 divided by precision, also referred to as the "number needed to read". Additionally, we calculated 1-specificity (or "fall-out"), which represents the false positive rate and gives an indication of the probability that a non-relevant document is retrieved by a search. The formulas used to calculate these properties are summarized in Table 1.

**Table 1:** Formulas for calculating sensitivity, 1-specificity, and precision.

	Eligible articles	Ineligible articles	Total articles
Retrieved by search strategy	a	b	a+b

Not retrieved by search strategy	c	d	c+d

Total	a+c	b+d	N

### Validation of search strategies

Because the same CRTs were used to both derive and evaluate the search strategies, we additionally tested search strategies against an independent set of 363 CRTs that had been identified in seven previously published systematic reviews of CRTs. The year of publication for these trials ranged from 1979 to 2005. We obtained UI numbers for these trials and determined the % retrieved by the search strategies ("relative recall" [17]).

### Trends in reporting standards

To determine whether the reporting of CRTs has improved over time, we calculated the percentage of trials clearly identified as "cluster randomized", "group randomized", or "community randomized" in the title or abstract, and compared these percentages by year of publication.

## Results

### Existing search strategy for randomized controlled trials

The number of articles retrieved, as well as sensitivity, 1-specificity, and precision of each search strategy is presented in Table 2. The existing search strategy for randomized controlled trials yielded sensitivity 93.8% and precision 9.0% (number needed to read 11) for identifying CRTs.

**Table 2:** Properties of search strategies evaluated against the gold standard set of CRTs

Search Strategy	Total articles retrieved	CRTs retrieved (Sensitivity %) (N = 162)	Non-CRTs retrieved (1-Specificity %) (N = 25545)	Precision
**Existing strategy for randomized controlled trials:**				
1. randomized controlled trial.pt.				
2. animals/				
3. humans/				
4. 2 NOT (2 AND 3)				
5. 1 NOT 4	1697	152 (93.8%)	1545 (6.0%)	9.0%

**Cluster design-related terms:**				
6. cluster$ adj2 randomi$.tw.	87	59 (36.4%)	28 (0.11%)	67.8%
7. ((communit$ adj2 intervention$) OR (communit$ adj2 randomi$)).tw.	96	16 (9.9%)	80 (0.31%)	16.7%
8. group$ randomi$.tw.	34	9 (5.6%)	25 (0.10%)	26.5%
9. 6 OR 7 OR 8	208	78 (48.1%)	130 (0.51%)	37.5%

10. intervention?.tw.	2636	139 (85.8%)	2497 (9.8%)	5.3%
11. cluster analysis/	150	30 (18.5%)	120 (0.47%)	20.0%
12. health promotion/	796	38 (23.5%)	758 (3.0%)	4.8%
13. program evaluation/	560	36 (22.2%)	524 (2.1%)	6.4%
14. health education/	428	32 (19.8%)	396 (1.6%)	7.5%
15. 10 OR 11 OR 12 OR 13 OR 14	3658	149 (92.0%)	3509 (13.7%)	4.1%
16. 9 OR 15	3680	155 (95.7%)	3525 (13.8%)	4.2%

**Highest sensitivity:**				
17. 16 OR 5	4583	161 (99.4%)	4422 (17.3%)	3.5%

**Highest precision:**				
18. 16 AND 5	794	146 (90.1%)	648 (2.5%)	18.4%

"$" allows for truncation of words so that variations such as "randomization", "randomisation", "randomized" are included; adj refers to the adjacency operator to accommodate terms such as "community-based randomized trial"; pt refers to publication type; ? refers to optional wildcard character retrieving 1 or 0 characters;/refers to MeSH; tw refers to text words in the title and abstract.

### Cluster design related search strategies

Analysis of the specific text from each trial that identified the trial as cluster randomized or possibly cluster randomized revealed that 78 (48.1%) of the gold standard set had been clearly identified as "cluster randomized", "group randomized" or "community randomized" in the title or abstract; however, 38 (23%) could be identified as cluster randomized only by manual inspection of the trial procedures in the full text article. The remaining 46 trials (28%) could be identified as cluster randomized or *possibly *cluster randomized based on the units of randomization (e.g., "schools were randomized", "patients were randomized by physician", "randomization by practice") (see Additional file 2: Examples of text in title or abstract suggesting trial as possibly cluster randomized). Electronic search strategies based on the units of randomization were examined but found to be infeasible. For example, search strategies for text words involving "hospitals were randomly assigned" were explored that combined units of randomization with random allocation using an adjacency operator, but such searches had low precision because they did not eliminate individually randomized trials in which "*patients in *the participating hospitals were randomly assigned". Increasing the adjacency distance beyond 2 was explored but was found to be infeasible because of very low precision. Secondly, the cluster unit did not always appear in close proximity to the reference to random assignment (e.g., "High schools (N = 24) paired on enrolment size, racial composition, urban or rural location, and class structure were randomized"). Finally, it would be difficult to anticipate in advance all possible units of randomization that could be used in the diverse settings in which CRTs are implemented (e.g., football teams, churches, public housing complexes, pubs, swimming pools, or "balozi" (household clusters)). Cluster design related search strategies were therefore developed using primarily the frequency analyses of the MeSH and other text words in the title or abstract.

The strategy with highest sensitivity (Table 2 line 17), combined cluster design-related search terms with the existing strategy for randomized controlled trials using the Boolean operator OR; it retrieved 4583 articles, yielding sensitivity 99.4%, and precision 3.5% (number needed to read 28.6). The strategy with highest precision (Table 2 line 18) combined the cluster design-related search terms with the existing strategy for randomized controlled trials using the Boolean operator AND; it retrieved only 794 articles, yielding sensitivity 90.1%, and precision 18.4% (number needed to read 5.4).

### Validation of search strategies

The results of the search strategies evaluated against 363 studies included in previous systematic reviews are presented in Table 3. The relative recall of the existing strategy was virtually identical to that in the gold standard set, but the relative recall values of the cluster design-related strategies were lower, namely 97.8% and 80.7% for the most sensitive and most precise strategies respectively.

**Table 3:** Relative recall of search strategies to identify trials included in previous systematic reviews

					Number (%) of trials retrieved		
**Publication**	**Setting**	**Review Period**	**Sources searched**	**# Trials**	**Simple strategy**	**Taljaard highest sensitivity**	**Taljaard highest precision**

Donner et al. [5]	CRTs of non-therapeutic interventions	Jan 1979 -Aug 1989	The Lancet, NEJM, AJE, International Journal of Epidemiology	16	16 (100%)	16 (100.0%)	8 (50.0%)

Simpson et al. [6]	CRTs in primary care	1990 - 1993	AJPH, Preventive Medicine	21	15 (71.4%)	20 (95.2%)	15 (71.4%)

Eldridge et al. [9]	CRTs in primary care	1997, 2000	CENTRAL	145	140 (96.6%)	142 (97.9%)	121 (83.4%)

Isaakidis et al. [10]	CRTs in sub-Saharan Africa	Before Nov 2001	MEDLINE, CENTRAL, African Published Trials Register	51	45 (88.2%)	48 (94.1%)	33 (64.7%)

Puffer et al. [7]	CRTs	Jan 1997 -Oct 2002	BMJ, Lancet, NEJM	36	36 (100%)	36 (100%)	33 (91.7%)

Varnell et al. [8]	Group randomized trials	Jan 1998 -Dec 2002	AJPH, Preventive Medicine	60	54 (90.0%)	59 (98.3%)	51 (85.0%)

Eldridge et al. [11]	CRTs in primary care	2004 - 2005	BMJ, BJGP, Family Practice, Prev Med, Ann Intern Med, JGen Int Med, Pediatrics	34	34 (100%)	34 (100%)	32 (94.1%)

**Total**				**363**	**340** **(93.7%)**	**355** **(97.8%)**	**293 (80.7%)**

Abbreviations: CRT = cluster randomized trial; CENTRAL = Cochrane controlled trials register; NEJM = New England Journal of Medicine; AJPH = American Journal of Public Health; AJE = American Journal of Epidemiology; BJGP = British Journal of General Practice; IJE = International Journal of Epidemiology; Prev Med = Preventive Medicine; Ann Intern Med = Annals of Internal Medicine; J Gen Int Med = Journal of General Internal Medicine.

### Trends in reporting

The results of our analysis of trends in adequate reporting are presented in Table 4. Although interpretation of these results is complicated by the small sample sizes in some years, there appears to be a trend towards improvement (χ^2 ^test for trend = 3.6; p = 0.0003). Because publication year 2006 (unlike the remainder of the years) had not been randomly allocated for searching but represented trials identified from 10 major medical journals, we repeated this analysis excluding the 2006 journals. The results were similar, indicating an improvement over time (p = 0.0012). The lowest percentage of trials clearly identified (1 of 16 trials or 6.3%) was in 2001; this percentage increased to above 50% for the first time in 2003 and remained above that level until 2007. Of the 58 trials published in the first four years (2000-2003), 27.6% were clearly identified; this percentage more than doubled in the second half of the observation period (2004-2007) with 59.6% of 104 trials clearly identified (absolute increase in proportions 32.0%, 95% confidence interval 17.2 to 46.9%).

**Table 4:** Frequency and percentage of trials in the gold standard set that were clearly identified as cluster randomized in the title or abstract, by year of publication.

Year of publication	Identified as CRT	Not identified	Total
2000	4 (28.6%)	10 (71.4%)	14

2001	1 (6.3%)	15 (93.8%)	16

2002	1 (11.1%)	8 (88.9%)	9

2003	10 (52.6%)	9 (47.4%)	19

2004	8 (53.3%)	7 (46.7%)	15

2005	13 (65.0%)	7 (35.0%)	20

2006	30 (57.7%)	22 (42.3%)	52

2007	11 (64.7%)	6 (35.3%)	17

Total	78 (48.1%)	84 (51.9%)	162

## Discussion

A growing number of studies are using the cluster randomized design to evaluate health care interventions [18]. Cluster randomized trials have unique features which require special considerations for appropriate design and analysis. Librarians, systematic reviewers, and methodologists interested in evaluating changes in the standards of conduct and reporting of CRTs, need efficient search strategies to identify CRTs in bibliographic databases such as MEDLINE. Existing electronic search strategies for identifying randomized controlled trials may yield acceptable sensitivity, but have low precision. We showed that precision can be improved through the addition of cluster design-related terms. Our strategy with highest precision (18.4%) combined the cluster design related terms with randomized controlled trial.pt using the Boolean operator AND, yielding sensitivity 90.1% and number needed to read 5.4. The strategy with highest sensitivity (99.4%) combined the cluster design related terms with randomized controlled trial.pt using the Boolean operator OR, but yielded lowest precision (number needed to read 28.6). This may limit its usefulness for systematic reviewers seeking to identify a representative sample of CRTs within a reasonable time period. As an example, we are currently conducting a systematic review of ethical issues in a representative sample of 300 CRTs in health research [19]. We estimated that it would require an average of 3 minutes per article retrieved to scan the abstracts and download the full text, where necessary, to confirm that the article is indeed reporting a CRT meeting our eligibility criteria. To reach our desired sample size, we would require 427 hours using the strategy with highest sensitivity, but only 81.6 hours using the strategy with highest precision.

One of the limitations of our study is that the trials which were used to derive the search strategies were not selected by random sampling, representing instead a judgement sample of journals likely to publish CRTs. This was necessary from a practical standpoint as manual searching of electronic journals is a time-consuming process and it was necessary to maximize the yield from these searches. Nevertheless, more than 25,000 articles were examined from a broad range of 78 journals believed to be representative of the disciplines in which these trials are being published. A second limitation is that the cluster design related search strategies were derived using subjective judgement, rather than more objective methods such as logistic regression techniques (e.g., [20]). This is suggested as an avenue for future research. Thirdly, our search strategies were evaluated in MEDLINE only. We expect our search strategies to have similar precision in other databases that include MEDLINE; however, because precision associated with any search strategy will vary with the prevalence of eligible articles in the database, precision of our search strategy may drop in databases that are less abundant in their inclusion of CRTs.

It is likely that any search strategy, when evaluated against the same set of studies that was used to derive the search strategy, would provide an over-optimistic view of its sensitivity. It is therefore important to validate a newly derived search strategy against an independent set of studies. We tested three search strategies against an independent set of 363 CRTs identified in previous systematic reviews, by calculating their relative recall. Although these systematic reviews varied somewhat in their focus (e.g., primary care settings only or non-therapeutic interventions only), they all used the same standard definition of a CRT and are therefore covered by the broader criteria of our search strategy, designed to identify all CRTs regardless of the setting. As expected, the relative recall values of our search strategies were lower when tested against the validation set. It should be noted, however, that the validation set included primarily older trials, ranging from a publication year of 1979 to 2005, whereas our search strategies were derived using trials published in the years 2000-2007 only. Moreover, our results have shown that appropriate reporting of trials as CRTs has improved over time. This is confirmed by the validation set: for example, the results in Table 3 show that the strategy with highest precision had a relative recall of 89.2% against the 130 trials included in the three most recent systematic reviews, as opposed to 76.0% against the 233 trials included in the earliest four systematic reviews.

The CONSORT statement extension to CRTs, published in 2004, recommended that authors clearly identify trials as cluster randomized in the title or abstract of reports. Although overall, fewer than half (48.1%) of trials included in the gold standard set had been identified as CRTs in titles or abstracts, there was a significant improvement over time with 60.7% of trials published post-CONSORT (2005-2007) clearly identified. This improvement cannot be solely attributed to CONSORT however, as increases in these proportions were evident even in the pre-CONSORT years; improvements are likely attributable to a combination of factors, including increased awareness of the unique characteristics of the clustered design promoted by several articles and books [e.g., [2]], as well as general improvements in standards of reporting randomized controlled trials after publication of the original CONSORT statement [14].

## Conclusions

Although the reporting of CRTs is improving, it is not yet adequate. Simple search strategies will not efficiently retrieve CRTs unless they have been appropriately tagged by MEDLINE indexers as randomized controlled trials. The NLM has, for over a decade now, been concentrating on correctly identifying and indexing randomized controlled trials in MEDLINE [13]. Specific reference to "cluster randomized trial" in the title or abstract of trial reports will allow database indexers to more easily identify and correctly index reports of randomized trials. We do not currently promote the introduction of a new publication type in MEDLINE for CRTs, as we believe that appropriate indexing of the publication type, together with the use of the phrase "cluster randomized" in the title or abstract will allow simple and efficient identification of that subset of trials in which the unit of randomization is at the cluster level. As authors and journal editors pay increased attention to clearly reporting the design in the titles and abstracts of reports, the sensitivity and precision of electronic search strategies presented in this article are expected to improve.

## Competing interests

The authors declare that they have no competing interests.

## Authors' contributions

MT led the data collection, development of the search strategies, data analysis, and interpretation of results and drafted the manuscript. JM participated in the development of the search strategies, data analysis and interpretation of results. JMG participated in the development of the search strategies and interpretation of results. AM participated in data collection. JCB, MPE and AD participated in interpretation of the results. All authors critically reviewed and edited the manuscript and all authors approved the final manuscript.

## Pre-publication history

The pre-publication history for this paper can be accessed here:

http://www.biomedcentral.com/1471-2288/10/15/prepub

## Supplementary Material

Additional file 1 **Subject categories and journals included in manual search to identify gold standard set of cluster randomized trials**. Subject categories and journals included in manual search to identify gold standard set of cluster randomized trials. Click here for file

Additional file 2 **Examples of text in title or abstract suggesting trial as cluster randomized**. Examples of text in title or abstract that could be used to identify the trial as cluster randomized or possibly cluster randomized. Click here for file
