# Supplementary material for: Electronic search strategies to identify reports of cluster randomized trials in MEDLINE: low precision will improve with adherence to reporting standards
Source: BMC Med Res Methodol. 2010 Feb 16;10:15. doi: 10.1186/1471-2288-10-15 (PMC2833170; doi:10.1186/1471-2288-10-15)
Supplement: Additional file 1 — Subject categories and journals included in manual search to identify gold standard set of cluster randomized trials. Subject categories and journals included in manual search to identify gold standard set of cluster randomized trials. [file 1471-2288-10-15-S1.DOC]

**Subject categories and journals included in manual search to identify gold standard set of cluster randomized trials[[1]](#footnote-2)**

| **JCR SCIENCE EDITION 2007** |
| --- |
| Dentistry, Oral Surgery and Medicine |
| Journal of Dental Research |
| Community Dentistry and Oral Epidemiology |
| Journal of public health dentistry |
| Education: Scientific disciplines |
| Medical Education |
| Emergency Medicine |
| Annals of Emergency Medicine |
| Endocrinology & Metabolism |
| Obesity Research |
| General and Internal Medicine |
| New England Journal of Medicine |
| Lancet |
| JAMA |
| PLoS Med |
| BMJ |
| Archives of internal medicine |
| CMAJ |
| American Journal of Preventive Medicine |
| Preventive Medicine |
| American Journal of Managed Care |
| British Journal of General Practice |
| Family Practice |
| Journal of Evaluation in Clinical Practice |
| Geriatrics and Gerontology |
| Age and Ageing |
| Health Care Sciences and Services |
| Medical Care |
| Medical Education |
| Health services research |
| Health policy and planning |
| Medical Decision making |
| Health promotion international |
| Journal of Public Health Policy |
| Journal of Evaluation in Clinical Practice |
| BMC health services research |
| Immunology |
| Aids |
| Infectious Diseases |
| Lancet Infectious Diseases |
| Aids |
| Sexually Transmitted Infections |
| Medical informatics |
| Medical Decision making |
| Journal of Evaluation in Clinical Practice |
| Nursing |
| Nursing research |
| Journal of advanced nursing |
| Midwifery |
| Public Health Nursing |
| Nutrition and Dietetics |
| The American Journal of Clinical Nutrition |
| Obesity Research |
| Public Health Nutrition |
| Oncology |
| Journal of the National Cancer Institute |
| Pediatrics |
| Pediatrics |
| Archives of Pediatrics and Adolescent Medicine |
| Journal of Adolescent Health |
| Public, Environmental and Occupational Health: |
| American Journal of Epidemiology |
| Bulletin Of The World Health Organization |
| International Journal of Epidemiology |
| Medical Care |
| American Journal of Public Health |
| American Journal of Preventive Medicine |
| Journal of Epidemiology and Community Health |
| Tobacco Control |
| Social Science & Medicine |
| Journal of Adolescent Health |
| Prevention Science |
| Tropical Medicine and International Health |
| American Journal of Tropical Medicine and Hygiene |
| Preventive Medicine |
| Annals of Epidemiology |
| Public Health Nutrition |
| Health expectations |
| Transactions Of The Royal Society Of Tropical Medicine And Hygiene |
| Journal of Medical Screening |
| Community Dentistry and Oral Epidemiology |
| Journal of Occupational Health |
| Injury Prevention |
| Health Education and Behavior |
| Patient Education and Counseling |
| American Journal of Health Promotion |
| Health Education Research |
| European Journal of Epidemiology |
| BMC Public Health |
| Health promotion international |
| Public health reports |
| Aids Education and Prevention |
| Journal of Public Health Policy |
| International Journal Of Occupational And Environmental Health |
| Canadian Journal of Public Health |
| American Journal of Health Behavior |
| Public Health Nursing |
| The Journal of School Health |
| Journal of public health dentistry |
| Psychiatry |
| Addiction |
| Rehabilitation |
| Clinical rehabilitation |
| Sport Sciences |
| Medicine And Science In Sports And Exercise |
| Substance Abuse |
| Addiction |
| Psychology Of Addictive Behaviors |
| Tropical Medicine |
| Tropical Medicine and International Health |
| American Journal of Tropical Medicine and Hygiene |
|  |
| **JCR SOCIAL SCIENCES EDITION 2007** |
| Education & Educational Research |
| Aids Education and Prevention |
| Health Education Research |
| The Journal of School Health |
| Health Policy & Services |
| Health expectations |
| Psychology, Clinical |
| Health psychology |
| Psychology, Multidisciplinary |
| Psychology Of Addictive Behaviors |
| Journal of Community Psychology |
| Social Sciences, Biomedical |
| Social Science & Medicine |
| Social Sciences, Interdisciplinary |
| Evaluation Review |
| Social Work |
| Journal of Community Psychology |
|  |
| **NOT LISTED IN JCR** |
| Journal of Community Health Nursing |
| Family planning perspectives |
| Journal of alcohol and drug education |
| Journal of public health medicine |

1. Some Journals appear in multiple categories [↑](#footnote-ref-2)
