# Supplementary material for: Electronic search strategies to identify reports of cluster randomized trials in MEDLINE: low precision will improve with adherence to reporting standards
Source: BMC Med Res Methodol. 2010 Feb 16;10:15. doi: 10.1186/1471-2288-10-15 (PMC2833170; doi:10.1186/1471-2288-10-15)
Supplement: Additional file 2 — Examples of text in title or abstract suggesting trial as cluster randomized. Examples of text in title or abstract that could be used to identify the trial as cluster randomized or possibly cluster randomized. [file 1471-2288-10-15-S2.DOC]

**Examples of text in title or abstract suggesting trial as cluster randomized**

| “church-based nutrition and physical activity intervention” |
| --- |
| “churches were randomly assigned” |
| “classes of 7th graders from 10 randomly selected schools were assigned” |
| “clinics, each with a minimum of 1500 continuously enrolled adolescent females, were randomly assigned” |
| “communities throughout the US were randomly assigned” |
| “Community-based health promotion intervention” |
| “Community-Level HIV Prevention Intervention” |
| “Educational programmes were given to bartenders (n =40) in a randomized design in six of 12 pubs” |
| “facilities matched for size were assigned randomly” |
| “health care units (N = 67) were randomized” |
| “health centres randomly assigned” |
| “offices of a large managed care organization in Colorado were randomly assigned” |
| “participating HCs were randomized” |
| “Practices were randomised” |
| “public housing complexes were randomly assigned” |
| “randomised clinical trial in 16 neighbourhoods (balozi) ” |
| “randomised controlled trial including 28 GPs” |
| “randomization by practice” |
| “randomization of the sample by elementary schools” |
| “randomized by physician” |
| “randomized controlled trial involving 24 community day schools” |
| “randomized controlled trial, involving all general practices in two primary care trusts” |
| “randomized trial at 28 swimming pools” |
| “randomly assigned by school” |
| “school (n = 16 elementary) as unit of random assignment and analysis” |
| “school districts were randomly assigned” |
| “school football teams (N = 1,506 players at baseline) in Oregon and Washington were assigned to” |
| “school was the unit of recruitment, assignment, and analysis” |
| “School-Based Exercise Intervention” |
| “school-based substance-abuse-prevention program” |
| “school-based suicide prevention program” |
| “school-based, randomized controlled trial” |
| “schools (764 children, mean age: 11.2 +/- 0.7 years) were randomly assigned” |
| “senior centers were randomly selected and assigned” |
| “Six general medicine teams from a 600-bed urban teaching hospital were randomly assigned” |
| “students were randomly assigned by classroom” |
| “surgeons were randomized” |
| “troops randomly assigned” |
| “unit of randomisation was the work site physician” |
| “villages were randomized” |
